
JOURNAL INFORMATION
==============================
NLM Title Abbreviation: J Diabetes Investig
Journal ID: J Diabetes Investig
NLM Journal ID: 101520702
Journal ID: JDI

Journal of Diabetes Investigation

ISSN: 2040-1116
EISSN: 2040-1124
Publisher: Wiley

ARTICLE INFORMATION
==============================
PMCID: PMC4906255
PMID: 27296856
DOI: 10.1111/jdi.12506
Article ID: JDI12506
Article version: 1
Subjects: The Asian Association for the Study of Diabetes; Abstracts from The 7th Scientific Meeting of the Asian Association for the Study of Diabetes and the Annual Scientific Meeting of the Hong Kong Society of Endocrinology, Metabolism and Reproduction, 21‐22 November 2015, Hong Kong, China; The Asian Association for the Study of Diabetes

The Asian Association for the Study of Diabetes

Electronic publication date: 2016 Jun 14
Print publication date: 2016 May
Volume: 7
Issue: Suppl 2
Issue title: Abstracts from The 7th Scientific Meeting of the Asian Association for the Study of Diabetes and the Annual Scientific Meeting of the Hong Kong Society of Endocrinology, Metabolism and Reproduction, 21‐22 November 2015, Hong Kong, China
First page: 3
Last page: 4
Copyright: © 2016 The Authors. Journal of Diabetes Investigation published by Asian Association for the Study of Diabetes (AASD) and John Wiley & Sons Australia, Ltd
License: This is an open access article under the terms of the Creative Commons Attribution License, which permits use, distribution and reproduction in any medium, provided the original work is properly cited.
License URL: https://creativecommons.org/licenses/by/4.0/

Page count: 2
source-schema-version-number: 2.0
component-id: jdi12506
cover-date: May 2016
details-of-publishers-convertor: Converter:WILEY_ML3GV2_TO_NLMPMC version:4.9.1 mode:remove_FC converted:14.06.2016

==============================
JDI Journal of Diabetes Investigation Open access
Official Journal of the Asian Association for the Study of Diabetes

### The Asian Association for the Study of Diabetes

### EXECUTIVE BOARD MEMBERS

Chair: Prof. Yutaka Seino
Vice Chair: Prof. Hong Kyu Lee
Vice Chair: Prof. Wenying Yang
Auditors: Mr. Seisuke Koumoto, Prof. Sung Rae Kim, Prof. Zhangrong Xu
Editor-in-Chief for JDI: Prof. Nigishi Hotta
Directors:
Prof. Lee-Ming Chuang
Prof. Nobuya Inagaki
Prof. Linong Ji
Prof. Weiping Jia
Prof. Takashi Kadowaki
Prof. Doo-Man Kim
Prof. Moon-Kyu Lee
Prof. Ronald Ma
Prof. Wayne H-H Sheu
Prof. Yukio Tanizawa
Dr. Alice Wong
Prof. Zhiguang Zhou
Endorsed by:
Chinese Diabetes Society
Chinese Taipei Diabetes Association
Hong Kong Society of Endocrinology, Metabolism and Reproduction
Japan Association for Diabetes Education and Care
Japan Diabetes Society
Korean Diabetes Association

### AFFILIATED SOCIETIES AND ASSOCIATIONS

Cambodian Diabetes Association
Chinese Diabetes Society
Chinese Taipei Diabetes Association
Diabetes Association of Thailand
Diabetes Hong Kong
Diabetes India
Diabetes Malaysia
Diabetic Society of Singapore
Endocrine Society of Sri Lanka
Hong Kong Society of Endocrinology, Metabolism and Reproduction
Indonesian Diabetes Association
Japan Association for Diabetes Education and Care
Japan Diabetes Society
Korean Diabetes Association
Macau Diabetes Association
Malaysian Diabetes Educators Society
Mongolian Diabetes Association
Myanmar Endocrinology and Metabolism Society
Philippine Diabetes Association
Taiwanese Association of Diabetes Educators
Vietnamese Association of Diabetes and Endocrinology

© 2016 The Authors. Journal of Diabetes Investigation published by
Asian Association for the Study of Diabetes (AASD) and John Wiley & Sons Australia, Ltd
This is an open access article under the terms of the Creative Commons Attribution License, which permits use, distribution and reproduction in any medium, provided the original work is properly cited.

### Committee of The 7th Annual Meeting of AASD

### ORGANIZING COMMITTEE

**Chair: Dr. June Li**
Co-Chair: Dr. Wing Yee So
Honorary Secretary: Dr. Ying Wai Ng
Honorary Treasurer: Dr. Andrea Luk

**Members:**
Dr. Cheung Hei Choi
Dr. Victor Hung
Dr. Siu Fung Lam
Dr. Emmy Lau
Dr. Jenny Leung
Prof. Ronald Ma
Dr. Jason Ng
Dr. Annette Tso
Dr. Yu Cho Woo
Dr. Chi Kin Yeung

### PROGRAMME COMMITTEE

**Chair: Prof. Ronald Ma**
Co-Chair: Prof. Kathryn Tan

**Members:**
Prof. Xueyao Han
Prof. Cheng Hu
Prof. Alice Kong
Dr. Calvin Lee
Dr. Hung Yuan Li
Dr. Soo Lim
Dr. Shih Yi Lin
Dr. Eun Jung Rhee
Dr. Peter Tong
Dr. Hironori Waki
Prof. Aimin Xu
Dr. Daisuke Yabe
Dr. Vincent Yeung

### INTERNATIONAL ADVISORY BOARD

**Chair: Prof. Karen Lam**

**Members:**
Prof. Juliana Chan
Prof. Lee Ming Chuang
Prof. Nobuya Inagaki
Prof. Linong Ji
Prof. Weiping Jia
Prof. Takashi Kadowaki
Prof. Kyung Soo Ko
Prof. Ki Up Lee
Prof. Yutaka Seino
Prof. Wayne Sheu

© 2016 The Authors. Journal of Diabetes Investigation published by
Asian Association for the Study of Diabetes (AASD) and John Wiley & Sons Australia, Ltd
